# Species diversity and clinical relevance of nontuberculous mycobacterium isolated from pulmonary and extrapulmonary samples in southeastern Turkey, 2014 to 2023: A retrospective cross-sectional study

**DOI:** 10.1097/MD.0000000000043415

**Published:** 2025-07-18

**Authors:** Özge Alkan Bilik, Nida Özcan, Hadice Selimoğlu Şen, Erdal Özbek

**Affiliations:** a Department of Microbiology, Selahaddin Eyyubi State Hospital, Diyarbakir, Turkey; b Department of Microbiology, Medicine Faculty, Dicle University, Diyarbakir, Turkey; c Department of Chest Disease, Medicine Faculty, Dicle University, Diyarbakir, Turkey.

**Keywords:** Mycobacterium, *Mycobacterium simiae*, nontuberculous mycobacterium, pulmonary disease

## Abstract

Infection with nontuberculous mycobacterium (NTM) has been on the rise worldwide. Although the incidence rate of NTM infection has increased, little is known about its species diversity and clinical significance. Investigating the variety and load of NTM species in pulmonary and extrapulmonary clinical isolates from a community in southeast Turkey is the goal of this study. The prevalence of NTM and the clinical significance of pulmonary NTM (PNTM) are evaluated in this cross-sectional study. Matrix-assisted laser desorption ionization-time of flight mass spectrometry (MALDI-TOF MS) and multiplex PCR were used to identify NTM species. Between 2014 and 2023, a total of 30,539 clinical samples from 14,586 tuberculosis (TB)-suspected patients were analyzed. Among the 1159 mycobacterial isolates, 85.25% were identified as *Mycobacterium tuberculosis* complex (MTBC), while 14.75% were identified as NTM. Of the NTM isolates, 88.88% were pulmonary, while 11.11% were extrapulmonary. The majority of NTM isolates consisted of slow-growing species (67.84%). MTBC rate has decreased while the NTM rate has increased over the years. Of the 171 NTM isolates, identification was performed for 66 isolates. Overall, 16 different NTM species were identified. The most frequently isolated species were *Mycobacterium simiae* 28.78% (19/66), *Mycobacterium avium* complex 21.21% (14/66), and *Mycobacterium abscessus* complex 13.63% (9/66). Among the 66 patients with NTM isolation, the mean age was 39 (±23.10). On applying the American Thoracic Society/Infectious Disease Society of America (ATS/IDSA) criteria to determine the clinical relevance of the 61 patients with PNTM isolation, only 36.06% had nontuberculous mycobacterial pulmonary disease (NTMPD), and the majority were caused by *M. simiae* (40.9%). Chronic obstructive pulmonary disease was significantly more common in the American Thoracic Society criteria -positive group (22.7%, *P* = .004). Nodular bronchiectatic patterns occurred significantly more often in the American Thoracic Society criteria-positive group (77.3%, *P* <.001). A statistically significant correlation was determined between NTMPD and fatigue, although fatigue was not specific for a diagnosis of NTMPD. Cough was the predominant symptom. In conclusion, our findings indicate that the species diversity of NTM isolates in our region differs from that observed in other cities across Turkey. *M. simiae* emerged as the species with the highest isolation frequency and clinical significance.

## 1. Introduction

Mycobacteria that are not classified as “*Mycobacterium tuberculosis* complex (MTBC) and *Mycobacterium leprae”* are referred to as nontuberculous mycobacterium (NTM).^[1]^ The unique cell wall structure of mycobacteria, which contains high levels of mycolic acids, causes these organisms to exhibit acid-fast properties. This characteristic makes them resistant to conventional staining methods, but they can be detected using acid-fast staining techniques.^[2]^

Most NTMs are opportunistic pathogens commonly found in natural environments, particularly in soil, aquatic sources, drinking water systems, dust and aerosols.^[3]^ Both systemic immunosuppressive conditions, such as hematologic malignancy, immunosuppressive therapy, and HIV/AIDS (human immunodeficiency virus/acquired immune deficiency syndrome), and locally impaired immunity due to underlying lung disease increase the propensity to NTM infections.^[4]^ Recent findings have demonstrated that NTMs, once considered mere contaminants, infections in immunosuppressed and immunocompetent individuals can induce pulmonary and extrapulmonary infections.^[5,6]^ Apart from pulmonary involvement, NTM infections also commonly affect the skin, soft tissues, and lymphatic system.^[5]^

Based on their rates of growth in culture, NTM are classified as either slow-growing or rapid-growing. Rapid-growing mycobacteria are capable of forming colonies on subculture within 7 days or less. Slow-growing mycobacteria (SGM) necessitate over 7 days to develop mature colonies upon subculture.^[5]^ To date, more than 250 species or subspecies of NTM have been identified (https://lpsn.dsmz.de/genus/mycobacterium), many of which can produce disease in humans of all ages.^[6]^ A substantial increase in the number of clinically relevant species and the total number of mycobacterial species has occurred in recent years.^[5]^

NTM infections may present with nonspecific symptoms, exhibiting a spectrum of clinical manifestations from asymptomatic cases to severe or potentially fatal conditions.^[5]^ Symptoms and signs are frequently clinically and radiographically indistinguishable from those caused by *Mycobacterium tuberculosis*.^[5,7]^ Identifying NTMs at the species level is essential for the treatment, surveillance, and epidemiology of the disease, because of their high resistance to conventional antituberculosis drugs and different antibiotic susceptibilities different species.^[8]^

Infections associated with NTMs are considered a major factor in mortality and morbidity related to lung diseases. Isolates obtained from sterile body fluids or tissues are typically regarded as clinically significant; however, assessing the clinical significance of respiratory isolates presents more difficulties.^[5]^ As NTM is ubiquitous in drinking water, a major route of infection is through aerosols.^[3]^ Consequently, the presence of NTM in a respiratory specimen does not automatically signify the existence of disease.^[5]^ It is crucial to differentiate between transient or persistent colonization and infection in order to evaluate the clinical significance of a positive NTM culture obtained from a pulmonary sample.^[5]^ Microbiologic, radiographic, and clinical criteria are equally important, and all must be met for diagnosis of NTM pulmonary disease (NTMPD).^[5]^ To meet the diagnostic criteria for NTMPD, a patient must exhibit characteristic symptoms, have compatible radiological findings, and present with either 1 positive bronchial lavage, 2 or more positive sputum samples of the same NTM species, or compatible histopathological findings along with 1 positive culture. It is essential to exclude other potential causes of pulmonary disease.^[5]^

The diversity and pathogenicity of NTM species vary by geographical region.^[9]^ The prevalence of different NTM species also differs across continents, regions, and countries.^[10]^ Unfortunately, as in many regions worldwide, the reporting of NTM is not mandatory in our country; consequently, further investigation is needed to better define the disease burden, geographical distribution, and clinical relevance. Few studies have documented the frequency and species diversity of NTM in clinical samples,^[11]^ however, limited data exist on their role in clinical disease within our country. Therefore, in this study, we aimed to identify NTM species and assess their clinical relevance in pulmonary NTM (PNTM), while also analyzing the distribution of NTM in relation to patients’ demographic and clinical data.

We conducted a study of NTM isolates from pulmonary and extrapulmonary samples over a 10-year period (2014–2023) to evaluate their local epidemiology and clinical relevance.

## 2. Methods

### 2.1. Study design and patient selection

**Figure 1.**
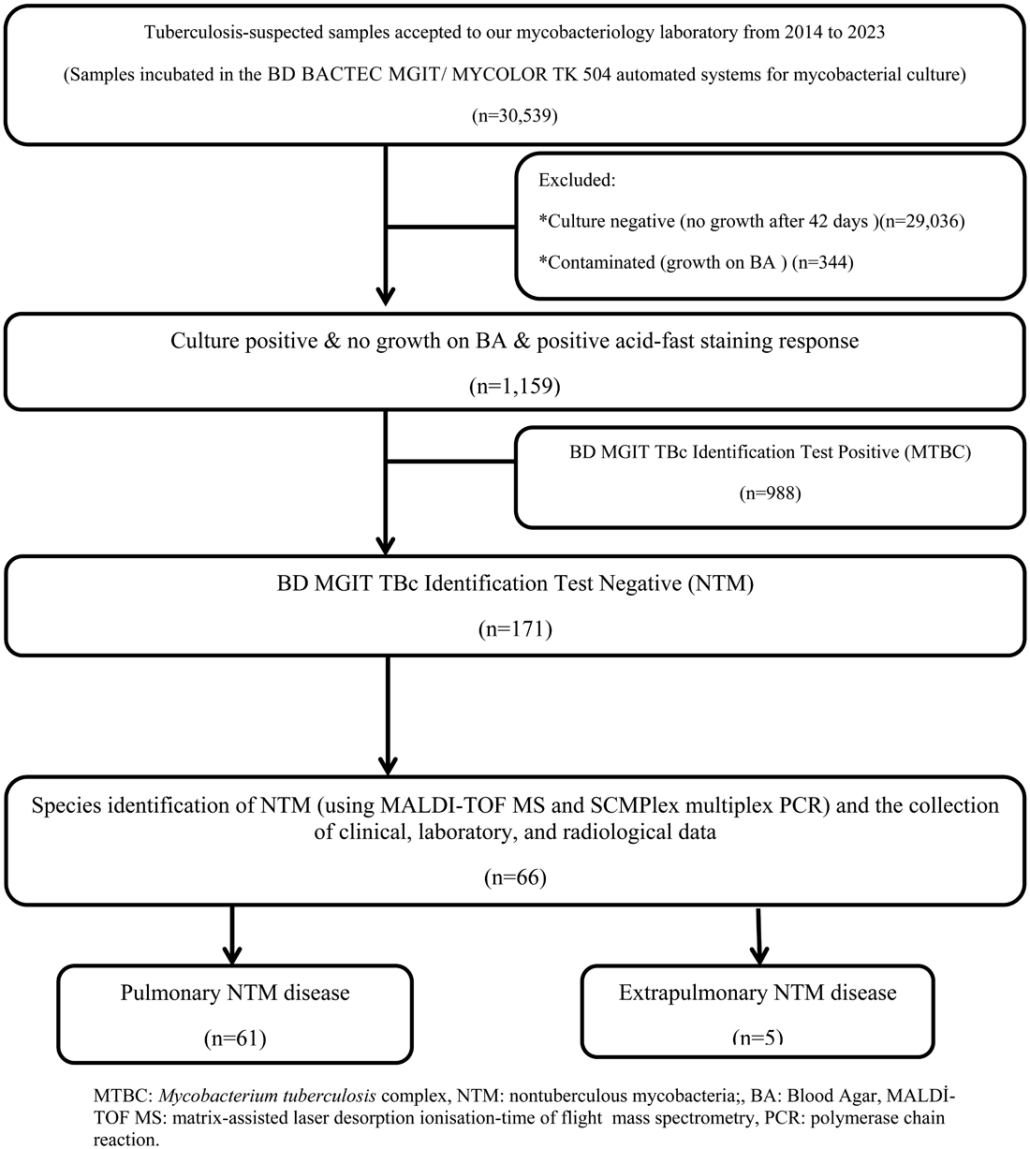
Flowchart of study design and patient selection. BA = blood agar, MALDİ-TOF MS = matrix-assisted laser desorption ionization-time of flight mass spectrometry, MTBC = *Mycobacterium tuberculosis* complex, NTM = nontuberculous mycobacteria, PCR = polymerase chain reaction.

We conducted a cross-sectional retrospective study on tuberculosis (TB)-suspected patients who sent their samples to the Mycobacteriology Laboratory at Dicle University Hospital between 2014 and 2023. Given that the Mycobacteriology Laboratory at Dicle University (located in Diyarbakir province in the southeast of Turkey) is the sole facility capable of performing mycobacterial culture and typing over a 10-year study period, the study encompasses data from all surrounding cities. This study included patients with at least 1 culture growing NTM. Patients were categorized into PNTM and extrapulmonary NTM (EPNTM) according to the source of their first NTM cultures. For study design and patient selection see Figure 1.

### 2.2. Culture procedure

The samples from non-sterile sites with commensal bacterial flora were subjected to homogenization and decontamination by the standard N-acetyl-L-cysteine and sodium hydroxide (NALC/NaOH) method. Sample smear microscopy was performed using the Ziehl–Neelsen (ZN) staining assay. Samples were cultured on both liquid and solid media. For solid culture, we used Lowenstein–Jensen culture media. Liquid mycobacterial cultures were performed using the BACTEC mycobacterial growth indicator tube 960 (BD, USA) and MYCOLOR TK 504 (TiboBio, Turkey) automated systems. Cultures not showing any growth after 42 days of incubation were considered negative. Positive liquid cultures were checked for purity by plating a drop from each medium onto a blood agar (BA) plate and incubating at 37°C for 24 to 48 hours to rule out false-positive results due to bacterial contamination. When culture growth was observed, and no contamination was detected on BA, slides were prepared from these samples, stained using the ZN method, and examined for acid-fast bacilli (AFB) under a microscope (Fig. 1).

### 2.3. Identification of NTM

NTM species were identified by matrix-assisted laser desorption ionization-time of flight mass spectrometry (MALDI-TOF MS) and multiplex polymerase chain reaction (PCR) methods. Multiplex PCR was performed by the ScmPlex TB/NTM detection kit (SCM Biotech Co. Turkey) using the Bio-Rad CFX96 Dx System (Bio-Rad Laboratories, Hercules) real-time PCR device. Mass spectrometry was performed by the Microflex LT MALDI-TOF MS (Bruker Daltonik GmbH, Bremen, Germany) device.

### 2.4. Data sources

Medical records of patients from whom NTM were isolated, including laboratory data (type of clinical sample, time to detection, microscopy results), demographic characteristics, clinical symptoms, comorbidities, predisposing conditions, and radiological findings, were reviewed. Laboratory data were obtained from the laboratory information management system and laboratory notebook records. Clinical records and radiographic information by computerized tomography were examined using the hospital information management system and patient files. Subsequently, the clinical relevance was assessed by the American Thoracic Society (ATS)/Infectious Disease Society of America diagnostic criteria.^[5]^

### 2.5. Classification of patients with PNTM isolation according to ATS/IDSA guideline criteria

The NTMPD patients were classified into 2 groups, ATS criteria (ATSC)-positive and ATSC-negative, based on microbiological, radiological, and clinical criteria recommended by the ATS/IDSA.^[5]^

### 2.6. Ethics statement

The study was conducted in accordance with ethical principles stated in the “Declaration of Helsinki” and approved by the Non-Interventional Ethics Committee of Dicle University (decision no: 34/2024, dated: 12.18.2024). As this was a retrospective study, the requirement for written consent was waived.

### 2.7. Statistical analysis

IBM SPSS V26.0 (IBM Corporation, Armonk) was used for statistical analysis of the study’s data. Also, G*Power 3.1.9.7 software (Universität Düsseldorf: Psychologie-HHU) was used. The study population was determined to be 66 with the G*power program by taking an effect size of 0.5, α = 0.05, and power (1-β) = 0.90. Descriptive statistical analyses were performed on all patients. Continuous and categorical variables were presented as means ± standard deviation and numbers (%). Fisher’s exact test or chi-square test was used to analyze between-group differences in categorical variables, if appropriate. Statistical significance was defined as a *P*-value of .05 or less.

## 3. Results

### 3.1. Frequency of NTM species by year

Between 2014 and 2023, a total of 30,539 clinical samples from 14,586 TB-suspected patients were analyzed. The first sample of each patient was evaluated. Positive mycobacterial cultures were detected in 7.94% (1159/14,586) of patients. The NTM and MTBC rates among 14,586 TB-suspected patients were found to be 1.17% (171/14,586) and 6.77% (988/14,486), respectively. It was observed that the MTBC rate has decreased while the NTM rate has increased over the years. The percentage of NTM in positive mycobacterial cultures differed over the years, from 7.03% in 2016 to 26.08% in 2022 (see more in Table 1).

**Table 1:** The distribution over time of the NTM and MTBC isolates.

Year	TB-suspected patients samples	Number of TB-suspected patient	Positive mycobacterial culture	MTBC	NTM	NTM rates in samples growing mycobacteria
	n	n	n (%)	N (%)	N (%)	%
2014	3469	1630	147 (9.01)	132 (8.09)	15 (0.92)	10.20
2015	3247	1396	141 (10.10)	126 (9.02)	15 (1.07)	10.63
2016	3467	1458	128 (8.77)	119 (8.16)	9 (0.61)	7.03
2017	2777	1332	143 (10.73)	127 (9.53)	16 (1.20)	11.18
2018	3271	1439	116 (8.06)	95 (6.60)	21 (1.45)	18.10
2019	3019	1725	111 (6.43)	90 (5.21)	21 (1.21)	18.91
2020	1805	891	76 (8.52)	67 (7.51)	9 (1.01)	11.84
2021	2261	1602	110 (6.86)	82 (5.11)	28 (1.74)	25.45
2022	2529	1048	92 (8.77)	68 (6.48)	24 (2.27)	26.08
2023	4694	2065	95 (4.60)	82 (3.97)	13 (0.62)	13.68
Total	30,539	14,586	1159 (7.94)	988 (6.77)	171 (1.17)	14.75

MTBC = *Mycobacterium tuberculosis* complex, NTM = nontuberculous mycobacterium, TB = tuberculosis.

### 3.2. Mycobacterial culture results

The study included 30,539 clinical samples from 14,586 patients, including pulmonary (n = 25,646) and extrapulmonary (n = 4893) specimens. Using the ZN staining method, 3.79% (1158/30,539) of the samples tested positive for AFB.

Among the 1159 mycobacterial isolates, 85.25% (988/1159) were identified as MTBC, while 14.75% (171/1159) were identified as NTM. Of the NTM isolates, 88.88% (152/171) were pulmonary, while 11.11% (19/171) were extrapulmonary. The majority of NTM isolates consisted of slow-growing species. The proportion of SGM was 67.84% (116/171), whereas the proportion of rapid-growing mycobacteria was 32.16% (55/171) (Table 2).

**Table 2:** Mycobacterial culture features.

	Frequency (n)	Percentage (%)
Total mycobacterial culture	30,539	–
AFB positivity	1158	3.79% (1158/30,539)
Culture-positive patient	1159	7.94% (1159/14,586)
MTBC	988	85.25% (988/1159)
NTM	171	14.75% (171/1159)
Pulmonary NTM	152	88.88% (152/171)
Extrapulmonary NTM	19	11.11% (19/171)
Slow-growing NTM	116	67.83% (116/171)
Rapid-growing NTM	55	32.16% (55/171)

AFB = acid-fast bacilli, MTBC = *Mycobacterium tuberculosis* complex, NTM = nontuberculous mycobacterium.

### 3.3. Species distribution and frequency of NTM species

**Figure 2.**
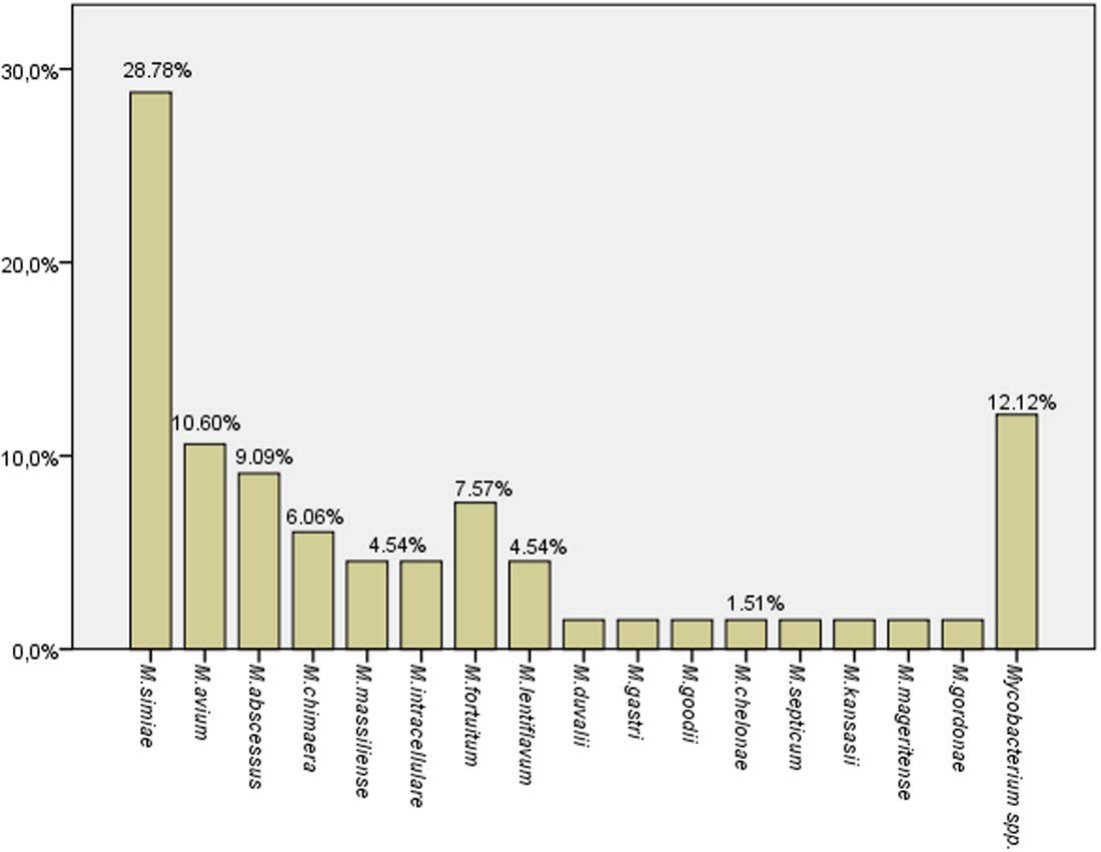
Distribution of the nontuberculous mycobacterial species (n = 66).

Of the 171 NTM isolates, speciation was performed for 66 isolates. Overall, 16 different NTM species were identified. The most frequently isolated species were *Mycobacterium simiae* 28.78% (19/66), *Mycobacterium avium complex* 21.21% (14/66), and *Mycobacterium abscessus* complex 13.63% (9/66). In the study, 12.12% (8/66) of NTM isolates could not be characterized at the species level. The distribution of the remaining NTM species, shown by frequency, is presented in Figure 2.

### 3.4. Distribution of NTM species in pulmonary samples

Of the NTM isolates, 92.42% (61/66) were identified as PNTM. Among the PNTM isolates, 88.52% (54/61) were obtained from sputum culture, 6.55% (4/61) from gastric aspirate, and 4.91% (3/61) from bronchoalveolar lavage (BAL). In the total of 61 pulmonary samples with NTM positivity, *M. simiae* was the most frequently isolated species, detected in 19 samples (17 sputum, 2 gastric aspirate). The distribution of NTM species in different pulmonary specimens is shown in Table 3.

**Table 3:** Distribution of NTM species in different pulmonary samples (n = 61).

	Sputum	BAL	Gastric aspirate	Total
*M. simiae*	17	0	2	19
*M. avium complex*				
* M. avium*	7	0	0	7
* M. chimera*	3	1	0	4
* M. intracellulare*	2	1	0	3
*M. abscessus complex*				
* M. abscessus*	5	0	0	5
* M. massiliense*	3	0	0	3
*M. fortuitum*	4	0	0	4
*M. lentiflavum*	2	1	0	3
*M. duvalii*	1	0	0	1
*M. gastri*	0	0	1	1
*M. goodii*	1	0	0	1
*M. chelonae*	1	0	0	1
*M. kansasii*	1	0	0	1
*M. gordonae*	0	0	1	1
*Mycobacterium* spp.	7	0	0	7
Total	54	3	4	61

BAL = bronchoalveolar lavage.

### 3.5. Distribution of NTM species in extrapulmonary samples

A total of 7.57% (5/66) of the NTM isolates were identified as EPNTM. Two of the EPNTM isolates were obtained from abscesses, 2 from tissue, and 1 from cerebrospinal fluid (CSF). The details are provided in Table 4.

**Table 4:** Distribution of NTM species in different extrapulmonary samples (n = 5).

	Abscess	Cerebrospinal fluid	Tissue	Total
*M. abscessus*	1	0	0	1
*M. septicum*	0	0	1	1
*M. fortuitum*	0	1	0	1
*M. mageritense*	1	0	0	1
*Mycobacterium* spp.	0	0	1	1
Total	2	1	2	5

NTM = nontuberculous mycobacterium

### 3.6. Demographic features and comorbidities of patients with NTM isolation

A total of 66 out of 171 NTM patients, for whom medical records were accessible, were analyzed for demographic features and comorbidities. Among the 66 patients with NTM isolation, the mean age was 39 (±23.10). A significant portion of the patients from whom NTM was isolated were younger than 40 years old.

Of the patients, 56.06% (37/66) were female, while 43.93% (29/66) were male. Regarding age distribution, 24.24% (16/66) were children (≤18), while 75.75% (50/66) were adults. The frequency of NTM isolation was 30.30% among patients in the age group 19 to 40 years, followed by 0 to 18 years at 24.24%. Diabetes mellitus was the most common (13.63%) comorbidity associated with the NTM infections. The other comorbidities were malignancy (12.12%), autoimmune diseases (9.09%), immunosuppressive therapies (9.09%), HIV/AIDS (1.51%), and congenital genetic disease (1.51%) (see more in Table 5).

**Table 5:** Demographic features and comorbidities of the patients with nontuberculous mycobacterium isolation (n = 66).

Features	n (%)
Age (mean ± SD)	39 ± 23.10
Gender	
Female	37 (56.06%)
Male	29 (43.93%)
Age group	
0–18	16 (24.24%)
19–40	20 (30.30%)
41–60	15 (22.72%)
61–80	13 (19.69%)
>80	2 (3.03%)
Risk factors	
Diabetes mellitus	9 (13.63%)
Malignancy*	8 (12.12%)
Autoimmune disease†	6 (9.09%)
Immunosuppressive therapy‡	6 (9.09%)
HIV (AIDS)	1 (1.51%)
Congenital genetic disease§	1 (1.51%)

HIV (AIDS) = human immunodeficiency virus/acquired immune deficiency syndrome, SD = standard deviation.

* Malignancies: breast cancer (n = 2), prostate cancer (n = 2), lung cancer (n = 1), gastric cancer (n = 1), histiocytic sarcoma (n = 1).

† Autoimmune diseases: systemic lupus erythematosus (n = 1), ankylosing spondylitis (n = 1), rheumatoid arthritis (n = 2), celiac disease (n = 1), Crohn's disease (n = 1), and myasthenia gravis (n = 1).

‡ Immunosuppressive therapies: Azathioprine (n = 2), Corticosteroid medicines (n = 4).

§ Primary ciliary dyskinesia.

### 3.7. Clinical relevance of NTM

Upon applying the ATS/IDSA criteria to determine the clinical relevance of NTM isolation in 61 patients with PNTM, only 36.06% (22/61) were diagnosed with NTMPD, with the majority caused by *M. simiae* (40.9%). These 22 patients met the full criteria for confirmed NTMPD and were classified as “ATSC-positive.” The remaining 64.93% (39/61) did not meet the ATS/IDSA diagnostic criteria and were classified as ‘ATSC-negative. Distribution of patient demographics, clinicals, laboratory and radiographic patterns are summarized in Table 6.

**Table 6:** Distribution of patient demographics, clinicals, laboratory, and radiographic patterns in 2 ATSC groups defined according to ATS/IDSA criteria (n = 61-pulmonary NTM).

Features		ATSC positive (n = 22)	ATSC negative (n = 39)	*P*-value
	–	n (%)	n (%)	–
Demography	Age (mean ± SD)	44.41 ± 20.90	35.54 ± 23.68	–
	Child	3 (13.6%)	11 (28.2%)	.225
	Adult	19 (86.4%)	28 (71.8%)	
	Female	9 (40.9%)	25 (64.1%)	.080
	Male	13 (59.1%)	14 (35.9%)	
Smoking status	Yes	7 (31.8%)	6 (15.4%)	.182
Sample species	Sputum	19 (86.4%)	35 (89.7%)	**.026** *
	BAL	3 (13.6%)	0	
	Gastric aspirate	0	4 (10.3%)	
AFB microscopy	AFB negative	16 (72.7%)	36 (92.3%)	**.038** **
	AFB positive	6 (27.3%)	3 (7.7%)	
NTM growing pattern	Slow growers	16 (88.9%)	23 (63.9%)	.053
	Rapid growers	2 (11.1%)	13 (36.1%)	
Symptoms	Cough	21 (95.5%)	34 (87.2%)	.795
	Dyspnoea	11 (50.0%)	18 (46.2%)	.617
	Haemoptysis	1 (4.5%)	6 (15.4%)	.196
	Fever	3 (13.6%)	6 (15.4%)	.592
	Night sweats	0	3 (7.7%)	.202
	Weight loss	3 (13.6%)	3 (7.7%)	.455
	Fatigue	3 (13.6%)	0	**.045** *
Immunosuppressive status	Yes	5 (22.7%)	13 (33.3%)	.383
Underlying lung disease	Yes	17 (77.3%)	20 (51.3%)	**.046** **
	Bronchiectasis	12 (54.5%)	16 (41.0%)	.309
	Asthma	4 (18.2%)	5 (12.8%)	.571
	COPD	5 (22.7%)	0	**.004** *
	Others	3 (13.5%)†	4 (10.4%)‡	.449
TB history	Yes	3 (13.6%)	6 (15.4%)	.853
Radiological pattern	Yes	22 (100%)	23 (59.0%)	**<.001** **
	Nodular bronchiectatic	17 (77.3%)	13 (33.3%)	**<.001** **
	Fibrocavitary	5 (22.7%)	11 (28.2%)	

The bold values mean significantly with a *P*-value < .05.

AFB = acid-fast bacilli, ATSC = American Thoracic Society criteria, BAL = bronchoalveolar lavage, COPD = chronic obstructive pulmonary disease, IDSA = Infectious Diseases Society of America, NTM = nontuberculous mycobacterium, SD = standard deviation, TB = tuberculosis.

† Interstitial lung disease (n = 1), cystic fibrosis (n = 1), pulmonary hypertension (n = 1).

‡ Bronşiolitis obliterans (n=1), lung cancer (n=1), cystic fibrosis (n=1), pulmonary hypertension (n=1).

* Calculated with Fisher's exact.

** Calculated with Pearson chi-square.

In the ATSC-positive group the mean age was higher (44.41 ± 20.90 years) compared to the ATSC-negative group (35.54 ± 23.68 years). Female patients constituted 40.9% of the ATSC-positive group and 64.1% of the ATSC-negative group. No statistically significant differences were observed between the groups in terms of age distribution or gender.

Sputum was the most common sample type in both groups. AFB microscopy positivity was higher in the ATSC-positive group (27.3%, *P* = .038). SGM isolates were more frequent in the ATSC-positive group (88.9%), whereas rapid growers were predominant in the ATSC-negative group (36.1%). The difference was approached but was not statistically significant (*P* = .053).

Cough was the predominant symptom in both groups (95.5% vs 87.2%). Fatigue was reported significantly more in the ATSC-positive group (13.6%, *P* = .045). Additional symptoms, including dyspnea, fever, hemoptysis, weight loss, and night sweats, exhibited no significant differences.

Underlying lung diseases were significantly more frequent in the ATSC-positive group (77.3%, *P* = .046). Chronic obstructive pulmonary disease was significantly more common in the ATSC-positive group (22.7%, *P* = .004).

Radiological abnormalities were detected in all ATSC-positive patients (100%) and 59.0% in the ATSC-negative group (*P* < .001). Nodular bronchiectatic patterns occurred significantly more often in the ATSC-positive group (77.3%, *P* < .001).

The clinical significance of the isolated species varied greatly among species. *M. simiae* was the most clinically relevant isolate and was the causative agent of NTMPD in 40.9% (9/22) of cases. The other clinically relevant isolates were *M. avium* (9.1%, 2/22), *M. chimerae* (9.1%, 2/22), *M. intracellulare* (9.1%, 2/22), *M. abscessus* (4.5%, 1/22), *M. lentiflavum* (4.5%, 1/22), and *M. fortuitum* (4.5%, 1/22).

## 4. Discussion

The number of NTM infections worldwide is increasing.^[12,13]^ The distribution of species among NTM isolates differs by continent and region.^[9]^ The data available in our country suggests that the number of studies that concentrate on the clinical relevance and distribution of NTM infections is relatively low.^[11]^ Despite the increasing prevalence and significance of NTM infections, the clinical relevance and comorbidities associated with these infections remain unclear. In this content, comprehensive studies are necessary. We aim to present local epidemiological data from our region and to enhance the literature regarding the clinical significance of NTMs. In this content, we wanted to document the current status and establish a reference framework for future studies. Our study provides comprehensive findings into the distribution, species diversity, and clinical relevance of NTM among TB-suspected patients.

This study identifies a notable trend: the increasing prevalence of NTM over time, alongside a declining rate of MTBC. The proportion of NTM in positive mycobacterial cultures rose from 7.03% in 2016 to 26.08% in 2022. This shift may be attributed to several factors, including improved diagnostic capabilities, greater clinician awareness, and potential environmental changes that promote NTM proliferation.^[5]^ Similar to the findings of this study, the prevalence of NTM increased globally over the years.^[11]^ The rising isolation rate of NTM in southeastern Turkey aligns with the increasing rates reported worldwide in recent decades.^[11,14]^ The number of NTM isolates significantly increased in the Middle East.^[15]^ Also, in Africa and the Middle East, the prevalence of NTM ranges from 4% to 15% among suspected TB cases.^[16]^ The frequency of NTM infection among TB-suspects was low (0.6%) in South India.^[17]^ In the current study, the NTM rate among 14,586 TB-suspected patients was found to be 1.17% (171/14,586). The decline in MTBC rates aligns with global tuberculosis control efforts, while the rising prevalence of NTM highlights the increasing clinical significance of these pathogens.

To enhance disease control, it is important to characterize NTM infection distribution by demographics or geographic region. However, results exhibit significant variability across studies, and the epidemiology of NTM infection varies among countries.^[18]^ To the best of our knowledge, this study is the first study to report the isolation frequency of NTM species from southeastern Turkey. The results of this study showed that *M. simiae* and *M. avium* were the most commonly isolated NTM species. This finding contrasts with observations from other regions in Turkey, where in previous studies *M. abscessus*, *M. lentiflavum*, and *M. fortuitum were* identified as the dominant species.^[11,19,20]^ In another study conducted in Istanbul, *Mycobacterium avium* complex, *M. abscessus, and M. kansasii* were the most common isolates.^[21]^ The rates of NTM disease exhibit significant variation based on geographical location.^[18]^ Our study reveals that the distribution of species varies significantly from region to region within our country.

In studies conducted worldwide, the most frequently isolated NTM were *M. intracellulare*, *M. avium*,^[22]^ *M. abscessus*,^[11,23]^ *M. fortuitum,*^[24]^ *M. kansasii,*^[12]^ while in our region, similar to Iran,^[25]^ Lebanon,^[26]^ and Ethiopia,^[27]^ the most frequently isolated NTM was *M. simiae. M. simiae* was traditionally thought to be restricted to the southern United States and Cuba.^[5]^ In a study conducted within the framework of the NTM-Network European Trials Group (NET), data on NTM isolation were collected from 20,182 patients across 30 countries, involving 62 laboratories, as of 2008. The study indicated that *M. simiae* was widely prevalent worldwide, except in Asia. However, a closer examination of the study revealed that the only Asian country participating in the research was Turkey, with laboratories located in western Turkey. Nonetheless, this observation does not fully represent the distribution of *M. simiae* across the entire Asian continent.^[9]^ Moreover, variations in isolation frequency are observed across different continents as well as among regions or cities.^[18]^ These disparities influence the local epidemiology of NTM infections, making awareness of the local context crucial for routine clinical practice.

The current study demonstrated that MTBC positivity was more dominant in mycobacterial cultures, and a significant proportion of NTM-positive cases were of pulmonary origin.

NTM represented 14.75% of the mycobacteria culture-positive isolates, predominantly consisting of slow-growing species (*M. simiae, M. avium*). These findings are consistent with prior studies that indicate a greater prevalence of MTBC in clinical samples, while also underscoring the rising significance of NTM.^[21,28]^ In our region, although MTBC remains the predominant mycobacterial pathogen among TB-suspected patients, the increasing prevalence of NTM infections—especially slow-growing species—highlights the need for improved diagnostic protocols to ensure effective patient management and treatment. This data may assist clinicians in selecting appropriate empirical antibiotics. In contrast to our country, developed countries such as Spain report a higher rate of NTM than MTBC.^[22]^

In this study, demographic analysis revealed a slight female predominance, consistent with previous studies,^[16]^ while the frequency of clinically significant NTM was higher in males. It is important to note that the average age of patients infected with NTM in our region is lower than that reported in other studies.^[22,29]^ Similar to our findings, NTMs were detected in younger age groups in Ethiopia, where *M. simiae* was the most frequently isolated NTM species.^[27]^

Applying the ATS/IDSA diagnostic criteria^[5]^ to assess clinical relevance revealed that 36.06% of patients had NTMPD, consistent with findings from prior studies conducted in Canada,^[30]^ the United Kingdom,^[23]^ and Gambia,^[10]^ while the rate was higher (60%) in some studies.^[31]^ The majority of clinically relevant NTM infections were caused by *M. simiae* (40.9%). Clinically significant NTM species vary across studies.^[23]^ The dominant NTM species in a geographic area might impact the clinically significant NTM species found in that region. Rare species, such as *M. duvalii*, *M. gastri*, *M. goodii*, *M. chelonae*, *M. kansasii*, and *M. gordonae*, were each isolated in a limited number of cases, highlighting the diversity of NTM species present in pulmonary samples. None of these rare species were clinically significant.

The higher mean age and prevalence of underlying lung diseases, such as chronic obstructive pulmonary disease, in the ATSC-positive group suggest that structural lung abnormalities significantly predispose individuals to NTMPD. This observation is in agreement with recent studies.^[29]^ Radiological abnormalities, particularly nodular bronchiectatic patterns on computerized tomography, were markedly more frequent in ATSC-positive patients, reinforcing their diagnostic importance. A statistically significant correlation was found between NTMPD and fatigue, although fatigue alone is not specific for a diagnosis of NTMPD. Cough was the predominant symptom, while additional symptoms, including dyspnea, hemoptysis, fever, night sweats, and weight loss, showed no significant differences. These findings may assist clinicians in better understanding the clinical features of patients with NTM.

## 5. Conclusion

In conclusion, the current findings indicate that the species diversity of NTM isolates in this region differs from that observed in other cities across Turkey. *M. simiae* emerged as the species with the highest isolation frequency and significant clinical relevance. The data shows an increase in the NTM isolation rate in this region, aligning with the global rise in NTM infections reported over the past decades. Enhanced awareness and targeted public health strategies are essential to address the growing burden of NTM infections in TB-endemic areas. Larger, multicenter studies are needed to comprehensively assess the full contribution of NTM to pulmonary disease.

## 6. Limitations

This study has some limitations. One of them is the inability to type all mycobacterial isolates using both mass spectrometry and PCR methods. Future studies could address this by utilizing sequencing techniques for the identification of these isolates. Additionally, the retrospective nature of patient data poses a risk of missing data, as some information may not have been recorded or may have been lost.

## Author contributions

**Conceptualization:** Özge Alkan Bilik, Nida Özcan, Hadice Selimoğlu Şen.

**Data curation:** Nida Özcan, Hadice Selimoğlu Şen.

**Formal analysis:** Özge Alkan Bilik, Nida Özcan, Hadice Selimoğlu Şen, Erdal Özbek.

**Investigation:** Özge Alkan Bilik.

**Methodology:** Özge Alkan Bilik, Nida Özcan, Hadice Selimoğlu Şen, Erdal Özbek.

**Supervision:** Özge Alkan Bilik, Nida Özcan, Hadice Selimoğlu Şen, Erdal Özbek.

**Visualization:** Özge Alkan Bilik.

**Writing – original draft:** Özge Alkan Bilik.

**Writing – review & editing:** Özge Alkan Bilik, Nida Özcan, Hadice Selimoğlu Şen, Erdal Özbek.
